# Intraoperative Performance of DaVinci Versus Hugo RAS During Radical Prostatectomy: Focus on Timing, Malfunctioning, Complications, and User Satisfaction in 100 Consecutive Cases (the COMPAR-P Trial)

**DOI:** 10.1016/j.euros.2024.03.013

**Published:** 2024-04-04

**Authors:** Alessandro Antonelli, Alessandro Veccia, Sarah Malandra, Riccardo Rizzetto, Vincenzo De Marco, Alberto Baielli, Andrea Franceschini, Francesca Fumanelli, Francesca Montanaro, Iolanda Palumbo, Greta Pettenuzzo, Luca Roggero, Maria Angela Cerruto, Riccardo Bertolo, Collaborators, Michele Aloe, Francesco Artoni, Paolo Bianchi, Claudio Brancelli, Sonia Costantino, Peres Fokana Pongmoni, Piero Fracasso, Giulia Marafioti Patuzzo, Antonio Raiti, Endri Toska, Vincenzo Vetro

**Affiliations:** Department of Surgery, Dentistry, Pediatrics and Gynecology, Urology Unit, University of Verona - Azienda Ospedaliera Universitaria Integrata Verona [AOUI], Verona, Italy; a Department of Surgery, Dentistry, Pediatrics and Gynecology, Urology Unit, University of Verona - Azienda Ospedaliera Universitaria Integrata Verona (AOUI) - Verona, Italy; b Residency Program in Health Statistics and Biometrics, University of Verona, Verona, Italy

**Keywords:** DaVinci, Hugo RAS, Robotic surgery, Radical prostatectomy, Surgical outcomes, Performance

## Abstract

**Background and objective:**

The Hugo RAS and DaVinci Xi systems are used for performing robot-assisted radical prostatectomy (RARP). This study aims to compare these two platforms providing granular and comprehensive data on their intraoperative performance.

**Methods:**

The Comparison of Outcomes of Multiple Platforms for Assisted Robotic surgery—Prostate (COMPAR-P) trial is a prospective post-market study (clinicaltrials.org NCT05766163). Enrollment began in March 2023, allocating patients to DaVinci or Hugo RAS for RARP, without selection criteria, for up to 50 consecutive cases. Two experienced console surgeons performed the procedures, following the same technique. Evaluation focused on timing, learning curves, malfunctioning events, complications, and users’ satisfaction, using standard statistical methods, including the cumulative summation analysis (CUSUM) for the learning curve assessment.

**Key findings and limitations:**

Fifty patients each were enrolled for DaVinci (DV-RARP) and Hugo RAS (H-RARP) RARP. Baseline features were balanced. DV-RARP showed significantly shorter “setup” and “console” phase durations than H-RARP (37 vs 55 min and 97 vs 126 min, respectively, *p* < 0.001). A longitudinal timing analysis revealed DV-RARP’s flat line, while H-RARP showed a modest decline with breakpoints at 22 and 17 procedures by CUSUM for the setup and console phases. The numbers of malfunctioning events were 4 (DV-RARP) and 20 (H-RARP). DV-RARP had high user satisfaction, while the user satisfaction of H-RARP varied. The comparison was between the first 50 H-RARP and the last 50 DV-RARP cases performed at our institution. This likely accounts for the observed differences in setup and console times between the cohorts. The specialized expertise of the surgeons involved could limit the generalizability of our findings.

**Conclusions and clinical implications:**

This prospective study compared unselected patients who underwent DV-RARP and H-RARP. More malfunctioning events occurred in case of Hugo RAS, but surgical outcomes were similar. Longer operative times for Hugo RAS were attributed to meticulous care with the novel platform. Improvement potential was evident within a few procedures, providing valuable insights for adopting this new platform.

**Patient summary:**

This study compared two advanced robotic systems, DaVinci and Hugo RAS, used to remove the prostate in patients diagnosed with prostate cancer. While both systems showed similar surgical outcomes, the newer Hugo RAS system required more meticulous movements, leading to slightly longer operation times. The findings suggest that, with further experience, both systems can provide effective treatment options for patients undergoing prostate surgery.

## Take Home Message

The present prospective study compared 50 versus 50 unselected patients who underwent robotic prostatectomy performed with the DaVinci versus Hugo RAS system. Although more malfunctioning/troubleshooting events were recorded during Hugo RAS cases, the surgery outcomes, including the occurrence of intraoperative complications, were not statistically different. Longer operative time was recorded for Hugo RAS cases, likely explained by the meticulous care applied in the surgery due to the use of the novel platform. Still, the prospect of improvement within a relatively low number of procedures was evident. All these findings can contribute to the adoption of this new platform.

## 1 Introduction

The DaVinci surgical system (Intuitive Surgical, Sunnyvale, CA, USA) has long been the gold standard in robotic-assisted surgery, dominating the field with its surgeon-friendly interface. Since its introduction, it has become synonymous with minimally invasive robot-assisted radical prostatectomy (RARP), known for its significant benefits.

After a 2-decade DaVinci monopoly, novel robotic platforms are now approved for clinical use. Such a striking event has generated the opportunity to open the market to competition with the expectation of reducing the costs of robotics [1]. Among the systems emerging as other frontrunners for urology in the European Community, the Hugo RAS system (Medtronic, Minneapolis, MN, USA) is gaining popularity. This platform has introduced peculiar features such as modular robotic arms and an open console architecture [2].

However, to date, this platform has shown uneven adoption across institutions and is still supported by limited scientific evidence, primarily comprising a few retrospective and single-arm studies. Notably, as of now, no planned prospective comparative trials have been published, but further evidence in this regard is eagerly awaited because an understanding of the nuanced differences between platforms, encompassing efficacy, ergonomics, cost effectiveness, and learning curves, likely represents a crucial issue for health care providers and patients alike [3].

Our institution promoted a prospective head-to-head comparison of newly introduced platforms with the “gold standard,” represented by the DaVinci system. The project, granted by the regional health care system, encompasses various surgical procedures across different specialties, including prostatectomy, partial nephrectomy, stomach, liver, pancreatic resections, colectomy, hysterectomy, etc. The “prostatectomy arm” of the project has recently been concluded; the present paper is focused on the comparison between the Hugo RAS and DaVinci Xi systems for RARP, with the aim to provide granular and comprehensive data on intraoperative performance of the platforms.

## 2 Patients and methods

The Comparison of Outcomes of Multiple Platforms for Assisted Robotic surgery—Prostate (COMPAR-P) trial is a monocentric, postmarket study promoted by the Azienda Ospedaliera Universitaria Integrata (AOUI) of Verona (Italy) within a broader project concerning different procedures and specialties [4]. The study received local ethical committee approval (4038CESC) and was registered on clinicaltrials.org (NCT05766163).

Enrollment began in March 2023. Unselected patients with organ-confined prostate cancer candidates to RARP at our department were allocated to one of the tested platforms, up to the completion of enrollment of 50 consecutive cases in each cohort. All patients signed informed consent: these documents were stored in a designated locker. The only exclusion criterion was the patient's refusal to consent to participate in the study.

The involved operative room personnel received dedicated intensive 3-d training at the ORSI Academy (Melle, Belgium). Two console surgeons (A.A. and V.D., with previous experience of >1000 and >500 DaVinci RARP [DV-RARP] interventions, respectively) performed the procedures. None of the surgeons carried out any procedure with the Hugo RAS before the recruitment for this study commenced. Three other surgeons, experienced in robotic assistance (A.V., R.R., and R.B.), were involved as table assistants. Anesthesiological protocol as well as patient positioning (supine position, legs joined and extended, and Trendelenburg 25°) was the same for DaVinci and Hugo RAS. Port placement, instead, varied according to the platform: for the DaVinci Xi system, four robotic trocars were put in line 2 cm above the umbilicus, plus two additional trocars (12 and 5 mm) triangulated to the robotic ones on the assistant side; for the Hugo RAS system, a W-shaped configuration was employed for the four robotic trocars, plus two additional trocars [5]. Equivalent additional resources were used across the two platforms concerning insufflation and aspiration systems, robotic instruments (monopolar scissor, Maryland bipolar forceps, fenestrated grasp, and needle driver), assistant instruments, sutures, and clips. No variation in surgical technique was implemented depending on the platform [5]. The laparoscopic approach was transperitoneal, using a 0° camera. An anterior antegrade dissection was performed, intra-, inter-, or extrafascially, according to clinical data; posterior and anterior reconstructions and anastomosis were done with barbed sutures. Lymph node dissection (LND) was indicated depending on the risk of lymph nodal invasion, as calculated by nomograms following an extended template [6].

The aim of the present study is the evaluation of the intraoperative performance of the DaVinci and Hugo RAS platforms, by assessing the following endpoints:

1. *Timing* and *learning curve*, as referred to the following phases of the procedure:
  (a) *Setup phase*, composed of the following steps: “room configuration,” “patient positioning,” “port placement,” “draping,” “docking,” “undocking,” and “undraping”
  (b) *Console phase*, composed of the following steps: “opening of the umbilical-prevesical fascia,” “preparation of the Retzius space,” “dissection of the bladder neck,” “dissection of the seminal vesicles,” “dissection of the posterior plane,” “management of prostatic pedicles and eventual nerve sparing,” “dissection of the prostate apex and urethra,” and “posterior reconstruction and urethrovesical anastomosis”; a separate comparison was done for LND
2. *Malfunctioning*, defined as any technical abnormality that persistently impaired the function of the robot; the events were detailed in terms of the type, duration, and management.
3. *Complications*, defined as any deviation from the regular intraoperative course of the intervention; the events were detailed in terms of the type and management, and graded according to the intraoperative adverse incident classification proposed by the European Association of Urology Ad Hoc Complications Guidelines Panel [7];
4. *Users’ satisfaction*, determined by a visual assessment scale (1—poor to 5—optimal):
  (a) First surgeon, assistant, and scrub nurse satisfaction concerning the whole procedure
  (b) First surgeon satisfaction with each robotic instrument
  (c) First surgeon satisfaction with the quality of the vision (image definition and depth perception), bimanual dexterity, instrument mobility (promptness, precision, and range of movements), force control, and usability of console commands

Deidentified data were prospectively collected in a Research Electronic Data Capture (REDCap) dataset during surgery by a dedicated investigator who was not directly involved in the procedure. The REDCap could be opened by a dedicated password-coded computer stored within a locker. Password was changed periodically and managed exclusively by the involved investigators.

### 2.1 Statistical analysis

The statistical analysis was performed according to guidelines [8]. The sample size was established according to study feasibility [4]. Both the Shapiro-Wilk test and the graphical assessment were adopted for assessing data distribution. The mean and standard deviation (SD), or the median and interquartile range (IQR) were used for reporting continuous variables based on data distribution. Frequencies and percentages were adopted for dichotomous data. In the case of normal distribution, the Student *t* test was chosen to evaluate the differences between the two groups, whereas the Mann-Whitney U test was deemed suitable for the nonparametric variables. The differences in the case of discrete variables were evaluated using Pearson’s χ^2^ test. To assess the learning curve for Hugo RAS, the single and overall times evaluated were reported graphically along the progression from the first to the 50th case, and compared with the corresponding DaVinci cases. Additionally, to define the learning curve for Hugo RAS, the cumulative summation analysis (CUSUM) was used. This method represents data from consecutive procedures, transforming raw data into a cumulative sum of differences between single values and the overall mean, described graphically by a curve: the breakpoint from the ascending to the descending portion indicates the number of cases when the transition from a learning to a proficiency phase occurs [9].

All the tests were two sided, with statistical significance set at *p* ≤ 0.05. Analyses were performed using STATA 18.0 (1996-2024; StataCorp LLC, Lakeway Dr, College Station, TX, USA). The following STATA syntax was adopted to run the tests: *swilk*, *histogram*, *ttest*, *tabstat*, *ranksum*, *tabulate*, *chi2*, and *column*. For the CUSUM estimation, Microsoft Excel 2023 (Microsoft 2024, Redmond, WA, USA) was used.

## 3 Results

Fifty and fifty patients were enrolled during the study period to be submitted to DV-RARP and RARP performed by the Hugo RAS (H-RARP) platform, respectively. Of interest, each of the involved console surgeons performed 25 cases with Hugo RAS.

The comparison of baseline features showed that groups were balanced, with differences limited to Charlson Comorbidity Index, cN+ rate—higher in the DV-RARP group, and Briganti (2018) score [6]—higher in the H-RARP group (Table 1).

**Table 1:** Baseline data

	Total 100	DaVinci 50	Hugo RAS 50	*p* value
Age at surgery (yr), mean (SD)	66.2 (5.7)	66.4 (5.5)	65.9 (5.9)	0.6
PSA baseline (ng/ml), median (IQR)	6.9 (5.2-9.3)	5.9 (4.8-8.7)	7.7 (5.9-11.0)	0.063
Previous abdominal surgery, *N* (%)	42 (42)	19 (38)	23 (46)	0.4
BMI (kg/m^2^), median (IQR)	26.2 (24.5-28.4)	27 (24.5-29.7)	25.4 (24.5-27.8)	0.2
Obese BMI ≥30, *N* (%)	14 (14)	10 (20)	4 (8)	0.07
CAD, *N* (%)	8 (8)	3 (6)	5 (10)	0.5
Diabetes	9 (9)	5 (10)	4 (8)	0.7
Hypertension	48 (48)	25 (50)	23 (46)	0.7
Preoperative continence	99 (99)	50 (100)	49 (98)	0.3
PCa familiar history	22 (22)	13 (26)	9 (18)	0.3
ASA score, *N* (%)				0.3
1	3 (3)	3 (6)	0	
2	77 (77)	38 (76)	39 (78)	
3	20 (20)	9 (18)	11 (22)	
Charlson Comorbidity Index				
1	1 (1)	0	1 (2)	<0.001
2	30 (30)	6 (12)	24 (48)	
3	37 (37)	18 (36)	19 (38)	
4	24 (24)	18 (36)	6 (12)	
5	5 (5)	5 (10)	0	
6	3 (3)	3 (6)	0	
Pooled IPSS-QoL score, median (IQR) Mild IPSS (1-7), *N* (%)	9 (5-15)40 (40)	8.5 (5-11)20 (40)	11 (5-17)20 (40)	0.2
Moderate IPSS (8-19), *N* (%)	48 (48)	25 (50)	23 (46)	
Severe IPSS (20-35), *N* (%)	12 (12)	5 (10)	7 (14)	
IIEF-5 score		(*n* = 49) *		0.5
Median (IQR)	19 (10-22)	18 (10-22)	20 (11-22)	
Normal (22-25), *N* (%)	32 (32.3)	15 (30.6)	17 (34)	
Mild ED (17-21), *N* (%)	33 (33.3)	15 (30.6)	18 (36)	
Mild-moderate ED (12-16), *N* (%)	13 (13.1)	7 (14.3)	6 (12)	
Moderate (8-11), *N* (%)	15 (15.2)	9 (18.4)	6 (12)	
Severe ED (5-7), *N* (%)	6 (6.1)	3 (6.1)	3 (6)	
PI-RADS no. 1, *N* (%)		(*n* = 44) *	(*n* = 40) *	<0.05
2	1 (1.2)	1 (2.3)	0	
3	16 (19)	12 (27.3)	4 (10)	
4	37 (44.1)	20 (45.5)	17 (42.5)	
5	30 (35.7)	11 (25.0)	19 (47.5)	
No. 1 PI-RADS side, *N* (%)		(*n* = 44) *	(*n* = 40) *	0.09
Right	46 (54.8)	20 (45.5)	26 (65)	
Left	37 (44)	24 (54.6)	13 (32.5)	
Bilateral	1 (1.2)	0	1 (2.5)	
No. 1 PI-RADS position, *N* (%)		(*n* = 44) *	(*n* = 41) *	<0.05
Apex	24 (28.2)	13 (29.6)	11 (26.8)	
Middle	39 (45.9)	25 (56.8)	14 (34.2)	
Base	20 (23.5)	4 (9.1)	16 (39)	
Apex middle	2 (2.4)	2 (4.6)	0	
cN, *N* (%)		(*n* = 40) *		<0.05
N0	82 (92.1)	33 (82.5)	49 (98)	
N+	8 (7.9)	7 (17.5)	1 (2)	
MRI prostate volume (cm^3^)		(*n* = 44) *	(*n* = 44) *	0.058
Median (IQR)	40 (31.5-53.5)	43 (35-57)	40 (29-50)	
ISUP biopsy, *N* (%)				0.3
1	24 (24)	15 (30)	9 (18)	
2	30 (30)	12 (24)	18 (36)	
3	33 (33)	19 (38)	14 (28)	
4	13 (13)	4 (8)	9 (18)	
Briganti (2012) ^[6]^		(*n* = 27) *	(*n* = 40) *	0.8
Median (IQR)	4 (2-9)	4 (2-9)	4 (2.1-11)	
Briganti (2018) ^[6]^		(*n* = 29) *	(*n* = 24) *	<0.05
Median (IQR)	7 (3-14.1)	4 (3-11)	10.7 (4.2-30.1)	
% Positive cores, median (IQR)	35.7 (20-53.3)	33.3 (26.7-53.3)	40 (17.4-53.3)	1

ASA = American Society of Anesthesiologists; BMI = body mass index; CAD = coronary artery disease; ED = erectile dysfunction; IIEF-5 = five-item version of the International Index of Erectile Function; IPSS = International Prostate Symptom Score; IQR = interquartile range; ISUP = International Society of Urological Pathology; MRI = magnetic resonance imaging; PCa = prostate cancer; PI-RADS = Prostate Imaging Reporting and Data System; PSA = prostate-specific antigen; QoL = quality of life; SD = standard deviation; * missing data.

**Fig. 1:**
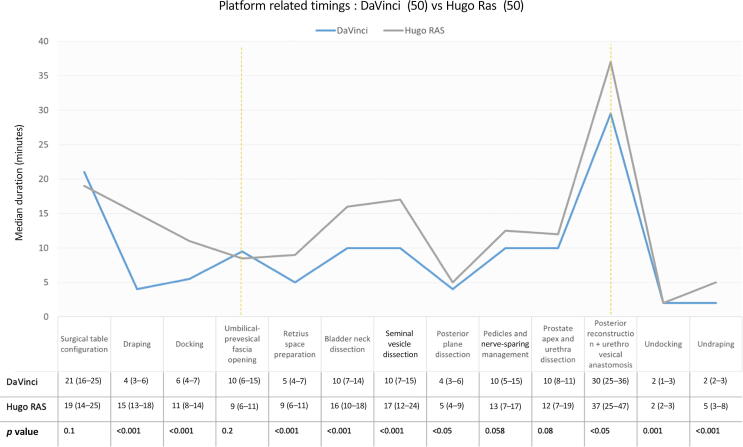
Segmentation of DaVinci- and Hugo RAS-related timing expressed as median duration. The setup phase is at the extremes of the yellow dashed lines.

The durations of the “setup” and “console” phases for the DV-RARP versus H-RARP group were 37 versus 55 min and 97 versus 126 min, respectively (both *p* < 0.001). A detailed analysis of the timing confirmed, for every single step of these two phases, a significantly lower duration in the DV-RARP group, except for “surgical table configuration,” “umbilical-prevesical fascia opening,” “pedicles and nerve-sparing management,” and “prostate apex and urethra dissection” (Fig. 1). LND was performed in 23 (47%) and 26 (52%) patients of the DV-RARP and H-RARP cohorts, with mean (SD) durations of 56 (18) and 56 (23) min, respectively (*p* = 1).

**Fig. 2:**
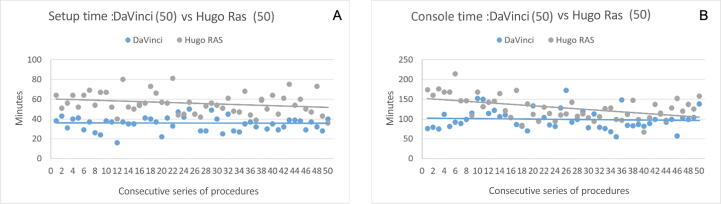
The median duration of the (A) setup and (B) console phases is shown for each procedure of the series (1-50).

**Fig. 3:**
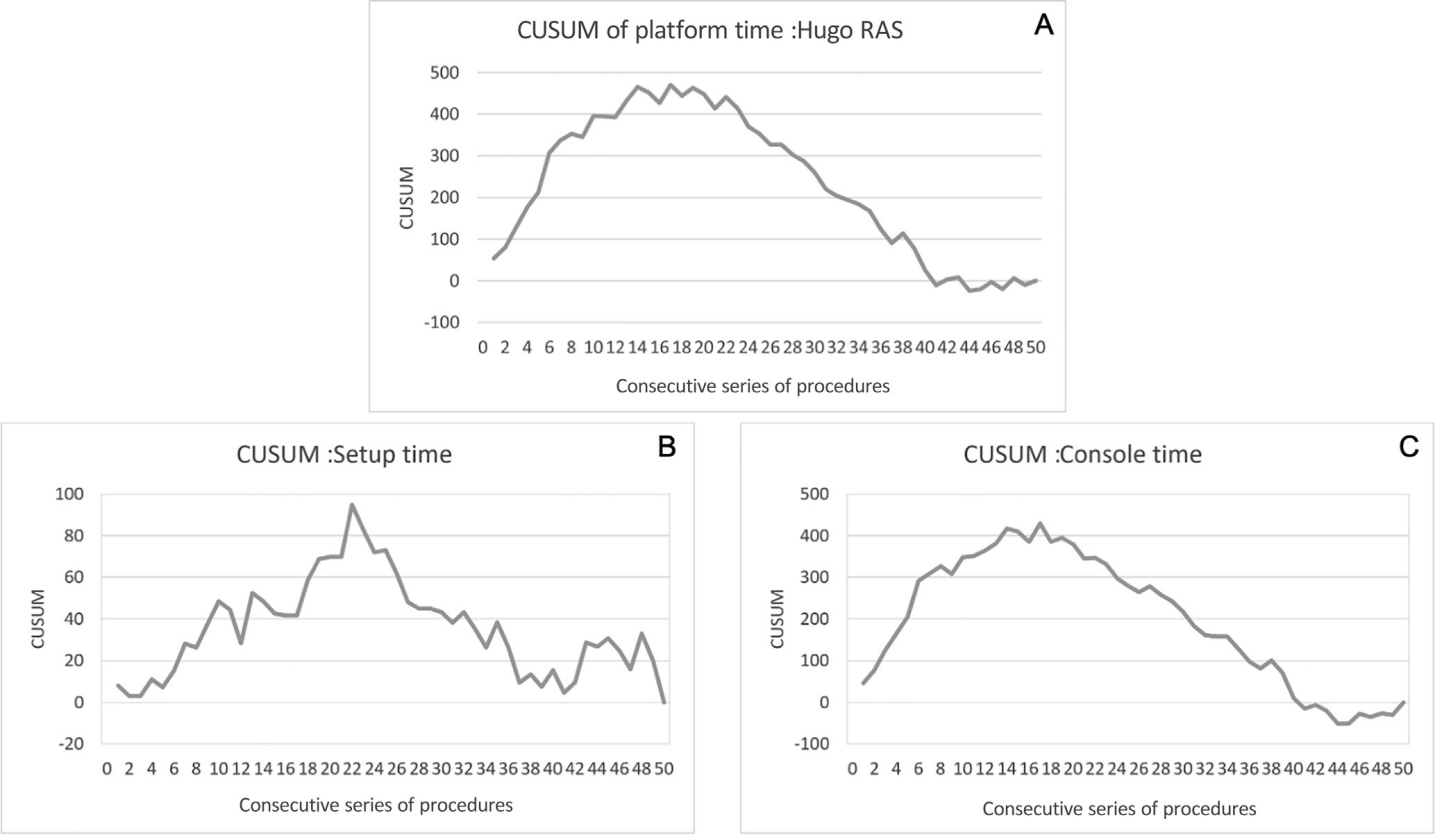
(A) CUSUM curve for the overall operative time (setup + console) of the Hugo RAS platform. Respective CUSUM curves of (B) setup and (C) console times are shown separately. CUSUM = cumulative summation analysis.

The graphical longitudinal analysis of timing—from the first to the 50th procedure—showed an almost flat line for the DV-RARP group, both for the setup and the console phase. Conversely, in the H-RARP series, the setup time duration declined modestly along with experience, while the console time duration declined markedly and tended to the DV-RARP values (Fig. 2; more details are provided in the Supplementary material). The analysis of the learning curve by the CUSUM graphs showed a breakpoint at 22 and 17 H-RARP procedures for the setup and console phases, respectively (Fig. 3).

Respectively, for the DV-RARP and H-RARP groups, four (time consumption 1-2 min) and 20 (time consumption up to 45 min) events of malfunctioning, as well as two and three events of intraoperative complications were reported (detailed in the Supplementary material). The median (IQR) estimated blood loss was 200 (150-300) and 300 (150-400) ml, respectively (*p* = 0.1).

**Fig. 4:**
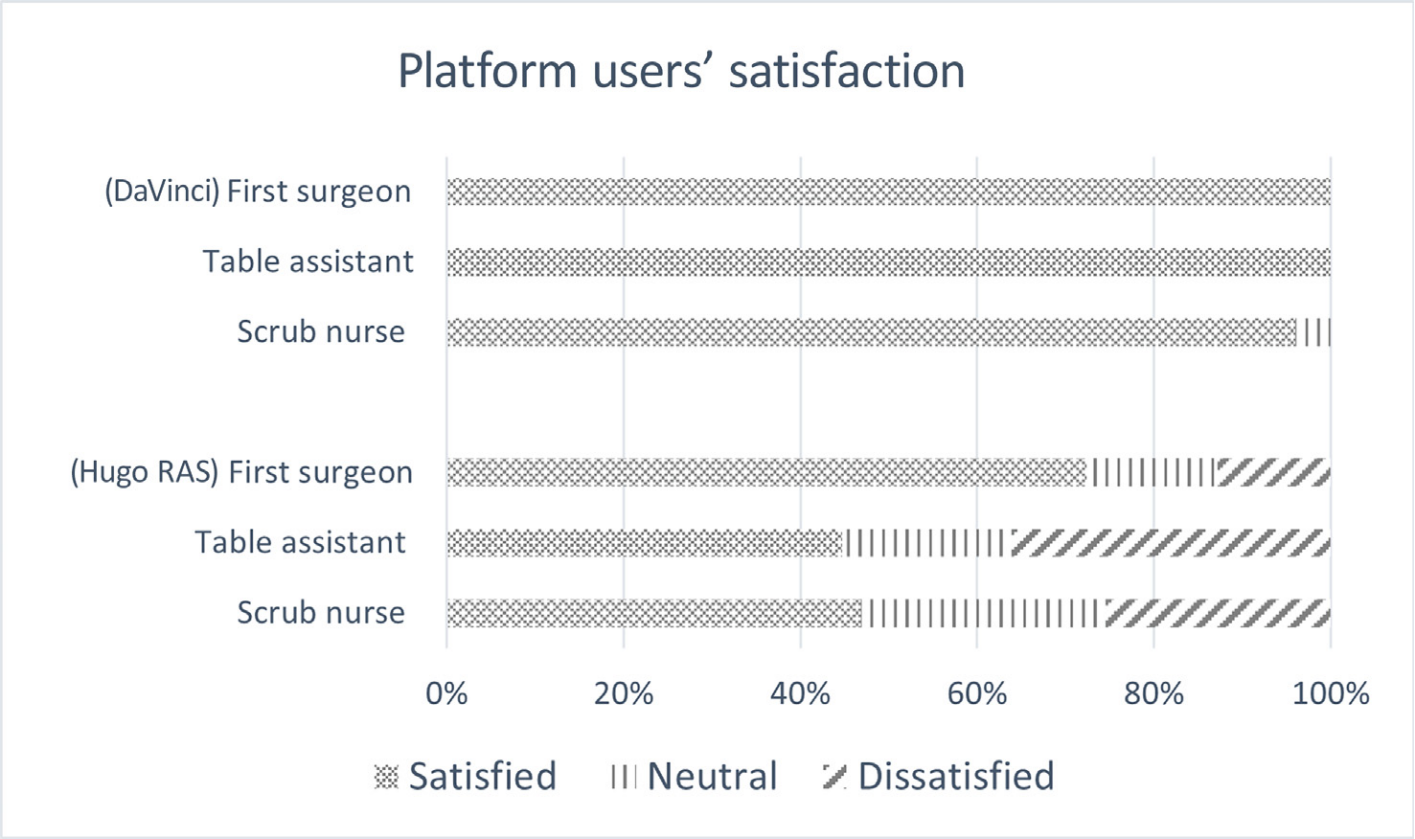
Users’ satisfaction (%), stratified by operative room personnel (first surgeon, table assistant, and scrub nurse) and platform (DaVinci vs Hugo RAS).

The evaluation of users’ satisfaction with DaVinci showed almost concordant complete satisfaction concerning overall performance, details on platform characteristics, and instruments. The overall satisfaction rates for Hugo RAS were instead 72%, 44%, and 47% for the first surgeon, assistant, and nurse, respectively; from 70% to 83% concerning the items describing the platform characteristics; and 72%, 89%, 64%, and 94% when evaluating the performance of “scissors,” “Maryland,” “Cadiere,” and “needle driver,” respectively (Fig. 4 and Supplementary material).

## 4 Discussion

The present study prospectively collected granular data to compare the DaVinci and Hugo RAS robotic platforms for performing RARP. It confirmed the expected difference in timing favoring the DaVinci system, both during the setup and the console phase. This can partly be related to the need to get confidence with a new “surgical instrument” but potentially also to the intrinsic differences between the platforms.

The graphical longitudinal analysis of the setup and console timing for DaVinci cases revealed an almost flat line, confirming that the team involved had well surpassed the learning curve. The same analysis for the Hugo RAS setup timing showed a mild decline along with experience, although it did not reach the efficiency level of DaVinci values. The breakpoint from learning to proficiency was identified in 22 cases. This finding suggests that the modular configuration with independent arms required additional efforts, particularly related to the need for separate draping/undraping and a more laborious docking process in terms of arms’ angles and port positioning.

The modest satisfaction indexes reported by the assistant surgeon and scrub nurse align reasonably with the additional work required, as well as the greater discomfort experienced due to the table side being “crowded” by the larger arms of the Hugo RAS. In a recent publication focusing on the perspectives of the bedside assistant, other authors emphasized the significance of her/his active role. This active involvement is crucial to ensuring the success of Hugo RAS pelvic procedures and preventing issues such as crushes, collisions, and other technological concerns that could negatively impact surgical performance [10].

In contrast, the decline in console times was steeper, with an earlier breakpoint set at 17 cases, and tended to overlap the DaVinci times toward the end of our experience. This indicates that once the system is docked, an expert surgeon can duplicate the surgical gestures adopted with the DaVinci with similar effectiveness.

However, it should be noted that this result also entails a certain degree of adaptation, as evidenced by the first surgeon's indicators of satisfaction regarding the overall performance of the platform, quality of vision and movement, and single robotic instrument. Although absolutely encouraging—and higher than the scores from the table personnel, these values were lower than those reported for DaVinci.

A crucial element to be considered is the significantly higher number of malfunctioning events recorded during Hugo RAS cases (20 vs 4). Remarkably, half of these events, including platform battery supply alarm, system power failure, conflict of arms, scissors rupture, malfunctioning of Maryland bipolar forceps, and failed calibration of the fourth arm, required over 5 min for resolution, significantly impacting the procedure flow and the total procedure time.

Overall, our data suggest that the new platform was “accepted” earlier by the console surgeon, while various “behind the scene” issues could complicate the assistance to RARP for other operating room personnel, despite their skill, training, and full dedication.

Our data align with previous experiences reporting that, for a surgeon with consistent DaVinci experience, skills are transferable soon from DaVinci to Hugo RAS when performing RARP and other pelvic procedures [11], [12], [13].

A few publications dealt with the preliminary experiences of RARP performed with Hugo RAS. The group headed by Dr. Mottrie described the first five RARP interventions performed with the Hugo RAS platform [2]. The authors reported a median console time of 120 min (IQR 110-150), which is very similar to that recorded from our experience. On the contrary, the authors judged both ports’ placement and docking as “straightforward and rapid”: we share the same perception based on our experience, but the more comprehensive assessment of the setup and the prospective comparison with DaVinci probably render our findings more solid. At the same time, the more intensive training done by this group [14], practicing in both dry and wet laboratory settings and testing the best operative setup on human cadavers, led to a faster reduction of some troubleshooting that we recorded in our series. It should be acknowledged that the training experienced by our group represents “real-life” conditions for most urologists. Still, we suggest that for the implementation of new technologies, mandatory laboratory-based training to proficiency should be considered, prioritizing patient safety over accepting such phenomena as reflective of real-life conditions. Other groups have published their very initial experience with H-RARP [15], [16], [17], but only Ou et al [18] described the need to find optimization in ports’ placement and docking. The authors observed some intraoperative issues that slowed the procedure flow. The intraoperative pauses for troubleshooting were mostly due to the mispositioning of the docking ports, which caused collisions of the robotic arms. Dr. Mottrie’s team followed with the publication of more consistent case series, including up to 112 patients, which confirmed the data reported at the start of the experience [19], [20]. Conversely, just a couple of papers attempted a comparison between the Hugo and DaVinci platforms, although in a retrospective manner. The first came from India, with 17 versus 17 patients compared, without finding significant differences [21]. The second, again from Mottrie’s group, analysed 378 versus 164 patients who underwent DV-RARP versus H-RARP [22]. The median total operative time was slightly longer for Hugo RAS procedures than for DaVinci surgeries—180 (IQR 150-200) versus 165 (IQR 130-200) min, a difference that was more pronounced in procedures without LND. Interestingly, our experience also confirms that LND did not exacerbate the differences between the platforms, resulting in comparable durations regardless of the robot used, likely because of the requirement of minimal assistance and instrument exchange. It is interesting to note that, with respect to the surgical learning curve for operative time with Hugo RAS, Mottrie and team did not find any association between increasing surgical experience and the time to complete surgery, possibly because the larger number of cases masked the impact of the learning curve or, alternatively, due to some bias in the recording of times related to the retrospective study design.

Nevertheless, despite the system's novelty at our institution and the technology disparity, it is crucial to emphasize that all surgeries were completed successfully within acceptable operative times and with comparable intraoperative complication rates to the fourth-generation DaVinci platform. This major finding confirms that, since its first generation, the Hugo RAS platform has the potential to be introduced rapidly into the clinical routine of a high-volume institution with an experienced team. Furthermore, we believe that some users could particularly appreciate some peculiar characteristics of the Hugo RAS platform, namely, the open console, different three-dimensional image quality of the console screen, sharpness of the monopoly shears, and efficiency of the monopolar coagulation. However, it is important to note that the user's perception of these features is highly subjective.

We acknowledge the main limitations of the present study, including the relatively small sample size and the limited generalizability of our findings. The study was underpowered to detect significant differences between treatment groups in some secondary endpoint outcomes, specifically intraoperative complications. This limitation is attributed to their relative rarity in the setting of a surgical trial conducted at a tertiary institution by expert surgeons.

We emphasize that the comparison was between the first 50 H-RARP cases and the last 50 DV-RARP cases performed at our institution. This distinction likely accounts for the observed differences in setup and console times between the treatment cohorts. While this aspect may be a common limitation in studies of this nature, we believe that our methodology remains robust, showcasing the strength of our overall effort. Furthermore, our results directly fit those of the surgeons experienced in DaVinci surgery who are contemplating a shift to the Hugo RAS platform. This scenario is likely to be one of the most common contexts for the adoption of this new robotic platform.

On the contrary, we underscore that the specialized expertise of the surgeons involved may restrict the direct applicability of the outcomes to settings with varying levels of surgical proficiency or experience. Introducing the Hugo RAS platform into a robotic-naïve center might present more challenges. To the best of our knowledge, there is a paucity of knowledge about this specific setting. As such, almost all the urological surgeons who started their experience with Hugo RAS had prior consistent experience with DaVinci robotic surgery. It is interesting to note that a cross-sectional study investigated surgeons of different robotic backgrounds who participated in a hands-on session with the Hugo RAS simulator (none of them had prior expertise with the system) and found that prior robotic console expertise improved basic skills at the Hugo RAS simulator. This undoubtedly has implications for skill transference across different platforms [23]. We strongly believe that, for centers/surgeons who are robotic surgery naïve, it would be advisable to invest in robust training programs, collaborate closely with experienced trainers or centers already using the Hugo RAS platform, and ensure a gradual integration process to optimize the transition.

## 5 Conclusions

The present prospective study compared 50 versus 50 unselected patients who underwent DA-RARP versus H-RARP. Although more malfunctioning/troubleshooting events were recorded during Hugo RAS cases, the outcomes of surgery, including the occurrence of intraoperative complications, were not statistically different. Longer operative time was recorded for Hugo RAS cases, likely explained by the meticulous care applied in the surgery due to the use of the novel platform. Still, the prospect of improvement within a relatively low number of procedures was evident. All these findings can contribute to the adoption process of this new platform.

  ***Author contributions*:** Alessandro Antonelli had full access to all the data in the study and takes responsibility for the integrity of the data and the accuracy of the data analysis.

  *Study concept and design*: Antonelli.

*Acquisition of data*: Veccia, Malandra, Rizzetto, De Marco, Baielli, Franceschini, Fumanelli, Montanaro, Palumbo, Pettenuzzo, Roggero.

*Analysis and interpretation of data*: Antonelli, Veccia, Malandra, Bertolo.

*Drafting of the manuscript*: Antonelli, Veccia, Bertolo.

*Critical revision of the manuscript for important intellectual content*: Antonelli, Cerruto, De Marco.

*Statistical analysis*: Veccia, Malandra.

*Obtaining funding*: Antonelli.

*Administrative, technical, or material support*: Malandra.

*Supervision*: Antonelli.

*Other*: None.

  ***Financial disclosures:*** Alessandro Antonelli certifies that all conflicts of interest, including specific financial interests and relationships and affiliations relevant to the subject matter or materials discussed in the manuscript (eg, employment/affiliation, grants or funding, consultancies, honoraria, stock ownership or options, expert testimony, royalties, or patents filed, received, or pending), are the following: None.

  ***Funding/Support and role of the sponsor*:** This work was supported by Regione Veneto (CRITE)—21/09/2022 rif. 434224 (prot. Gen. 56613/2022).

  ***Acknowledgments:*** The following authors contributed to the project as collaborators: Michele Aloe, Francesco Artoni, Paolo Bianchi, Claudio Brancelli, Sonia Costantino, Peres Fokana Pongmoni, Piero Fracasso, Giulia Marafioti Patuzzo, Antonio Raiti, Endri Toska, and Vincenzo Vetro (all from the Department of Surgery, Dentistry, Pediatrics and Gynecology, Urology Unit, University of Verona - Azienda Ospedaliera Universitaria Integrata Verona [AOUI], Verona, Italy).
