# Supplementary material for: Intraoperative Performance of DaVinci Versus Hugo RAS During Radical Prostatectomy: Focus on Timing, Malfunctioning, Complications, and User Satisfaction in 100 Consecutive Cases (the COMPAR-P Trial)
Source: Eur Urol Open Sci. 2024 Apr 4;63:104–12. doi: 10.1016/j.euros.2024.03.013 (PMC11000201; doi:10.1016/j.euros.2024.03.013)

**Supplementary Table 1.** Reporting on Technical Malfunctioning and Intraoperative Complications

| **Technical Malfunctions** | | **Action** | **Resolution time (minute)** |
| --- | --- | --- | --- |
| **Da Vinci^®^**  N = 4 | - Irreversible system failure (x2) - Failed instrument recognition - Cadiere forceps rupture | - Re-boot - Removal and re-connection - Substitution | - 1, 2 - 1 - 1 |
| **Hugo RAS^®^**  N = 20 | - Scissor cover-tip rupture (x3) - Platform battery supply alarm - System power on failure (x2). - Arms conflict (x2). - Failed instrument recognition (x4) - Scissor rupture (x5) - Maryland malfunctioning - Failed calibration arm#4 | - Substitution - Re-boot - Re-boot - Arms re-docking - Removal and re-connection - Manual removal and substitution - Substitution - Cable substitution and re-boot | - 2 - 45 - 10, 15 - 10, 6 - 2, 1, 1, 2 - 9, 4, 20, 20, 20 - 2 - 25, 30 |
| **Intraoperative Complications*** | |  |  |
| **Da Vinci^®^**  N = 2 | - Bladder wall injury - Small bowel perforation | - Repair (grade 1) - Mini-laparotomy and direct repair (grade 2) | - 2 - 91 |
| **Hugo RAS^®^**  N = 3 | - Bladder wall injury - Catheter entrapment during the suture for the anastomosis - Small bowel superficial injury due to cautery | - Repair (grade 1) - Liberation of the catheter (grade 0) - Suturing (grade 1) | - 4 - 2 - 32 |

*Graded according to the intraoperative adverse incident classification proposed by the European Association of Urology ad hoc complications guidelines panel [7]

**Supplementary Table 2.** Evaluation of Console Surgeon Satisfaction

| First surgeon evaluation  [scale 1 – 5] | **Dissatisfied**  [1 - 2] | | **Neutral**  [3] | | **Satisfied**  [4 - 5] | |
| --- | --- | --- | --- | --- | --- | --- |
| ***Platform Instruments***  Scissors  Maryland  Cadiere  Needle driver  ***Platform Characteristics***  Depth perception  Bimanual dexterity  Efficiency  Force sensibility  Robotic control | DaVinci^®^  0  0  0  0  0  0  0  0  0 | HugoRAS^®^  3  (6%)  2  (4%)  10  (21%)  1  (2%)  1  (2%)  2  (4%)  2  (4%)  2  (4)  1  (2%) | DaVinci^®^  0  0  0  0  0  0  0  3  (6%)  1  (2%) | HugoRAS^®^  10  (21%)  3  (6%)  7  (15%)  2  (4%)  7  (15%)  5  (11%)  12  (26%)  8  (17%)  11  (23%) | DaVinci^®^  33 (100%)  33 (100%)  33  (100%)  33  (100%)  48  (100%)  48  (100%)  48  (100%)  45  (94%)  47  (98%) | HugoRAS^®^  34  (72%)  42  (89%)  30  (64%)  44  (94%)  39  (83%)  40  (83%)  33  (70%)  37  (79%)  35  (74%) |

Satisfaction rate (%) of total provided responses.

**Supplement to Figure 2:**

1. The median overall operative time (set-up + console) duration is shown for each procedure of the series (1-50);


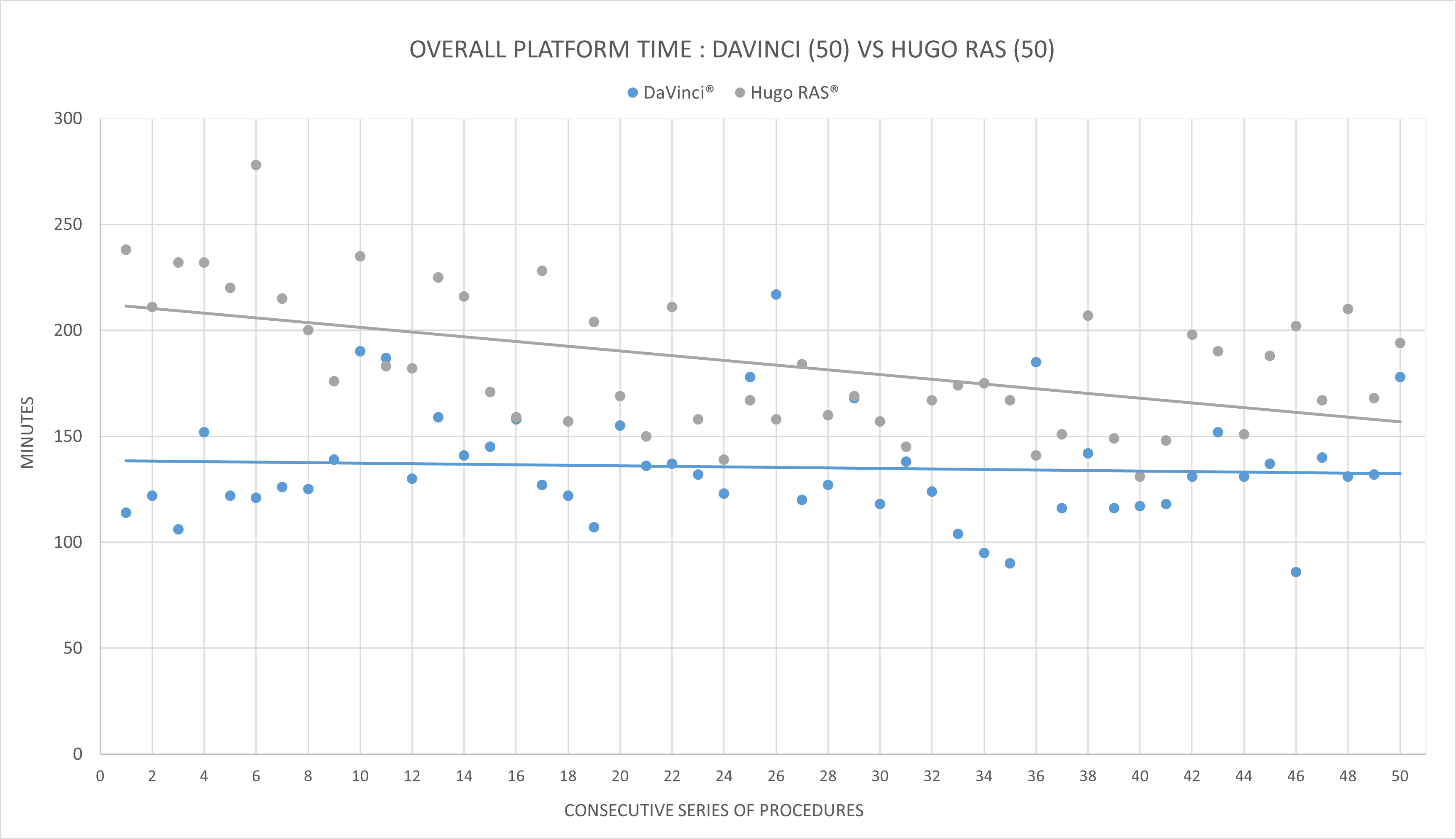


1. Each set-up phase is evaluated separately;


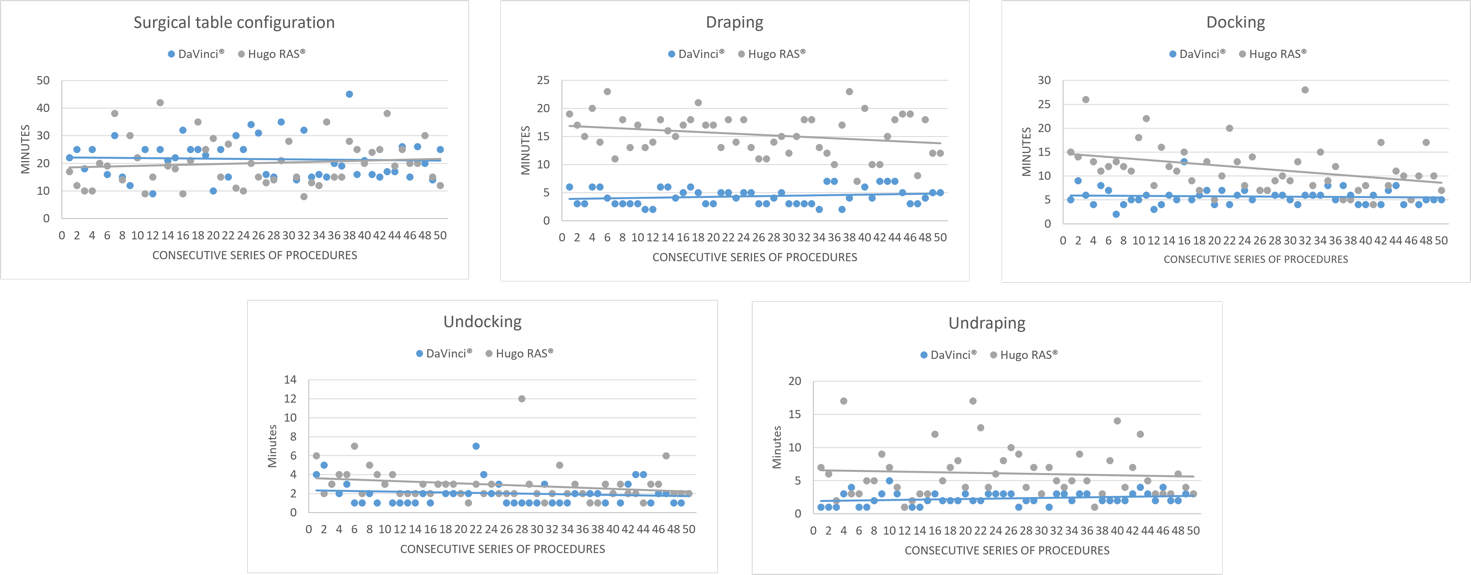


1. Each console phase is evaluated separately.


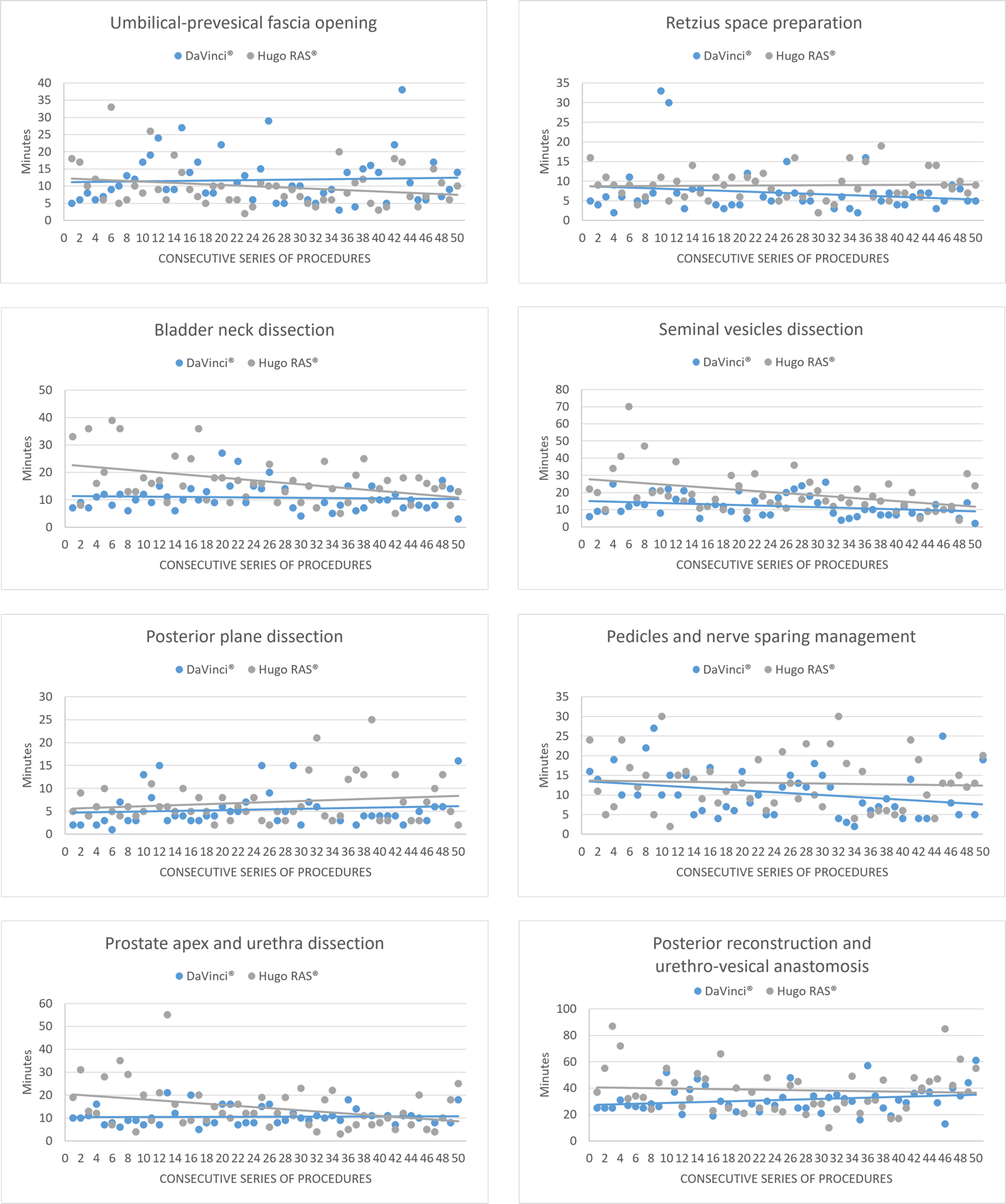

Supplement: Supplementary Data 1 [file mmc1.docx]
